# Interferon Regulatory Factor 4 Regulates the Development of Polymorphonuclear Myeloid-Derived Suppressor Cells Through the Transcription of c-Myc in Cancer

**DOI:** 10.3389/fimmu.2021.627072

**Published:** 2021-02-23

**Authors:** Quan Yang, Hongyan Xie, Xing Li, Yuanfa Feng, Shihao Xie, Jiale Qu, Anqi Xie, Yiqiang Zhu, Lu Zhou, Jinxue Yang, Xiaohao Hu, Haixia Wei, Huaina Qiu, Wenjuan Qin, Jun Huang

**Affiliations:** ^1^ The State Key Laboratory of Respiratory Disease, The First Affliated Hospital, Guangzhou Medical University, Guangzhou, China; ^2^ Sino-French Hoffmann Institute, Guangzhou Medical University, Guangzhou, China; ^3^ Department of Medical Oncology and Guangdong Key Laboratory of Liver Disease Research, The Third Affiliated Hospital, Sun Yat-Sen University, Guangzhou, China; ^4^ Department of Radiation Oncology, Zhongshan Hospital Affiliated, Xiamen University, Xiamen, China

**Keywords:** interferon regulatory factor 4 (IRF4), myeloid-derived suppressor cells (MDSCs), c-Myc, immunosuppression, cancer

## Abstract

The accumulation of myeloid-derived suppressor cells (MDSCs) is one of the major obstacles to achieve an appropriate anti-tumor immune response and successful tumor immunotherapy. MDSCs in tumor-bearing hosts are primarily polymorphonuclear (PMN-MDSCs). However, the mechanisms regulating the development of MDSCs remain poorly understood. In this report, we showed that interferon regulatory factor 4 (IRF4) plays a key role in the development of PMN-MDSCs, but not monocytic MDSCs. IRF4 deficiency caused a significant elevation of PMN-MDSCs and enhanced the suppressive activity of PMN-MDSCs, increasing tumor growth and metastasis in mice. Mechanistic studies showed that c-Myc was up-regulated by the IRF4 protein. Over-expression of c-Myc almost abrogated the effects of IRF4 deletion on PMN-MDSCs development. Importantly, the IRF4 expression level was negatively correlated with the PMN-MDSCs frequency and tumor development but positively correlated with c-Myc expression in clinical cancer patients. In summary, this study demonstrated that IRF4 represents a novel regulator of PMN-MDSCs development in cancer, which may have predictive value for tumor progression.

## Introduction

The immunosuppressive state of individuals with tumors is a key factor in limiting the body’s anti-tumor immune response. Immunosuppressive cells, including tumor-associated macrophages, marrow-derived suppressor cells, tumor-associated neutrophils, cancer-associated fibroblasts, and regulatory T cell interactions to actively promote tumorigenesis (1). Myeloid-derived suppressor cells (MDSCs) has well known roles in the suppression of anti-tumor immunity in tumor-bearing hosts (2, 3). Therefore, the key to anti-tumor immunotherapy is to design targeted therapy for the tumor immunosuppression mechanism, and targeting MDSCs has become a promising strategy for tumor immunotherapy (4, 5).

Mouse MDSCs, characterized by the co-expression of the myeloid markers CD11b and Gr1, are broadly classified into two distinct subsets, polymorphonuclear (PMN-MDSCs) and monocytic (M-MDSCs), based on the expression status of the Ly6G and Ly6C epitopes (6, 7). MDSCs are now defined as different subpopulations with specific phenotypes in human with clear immunosuppressive capacities, which have three subsets: M-MDSCs (HLA-DR^-^CD11b^+^CD33^hi^), PMN-MDSCs (HLA-DR^-^CD11b^+^CD33^low^), and e-MDSC (Lin^-^HLA-DR^-^CD33^+^) (8, 9). These subsets differ with respect to their function, tissue distribution, and regulatory mechanism (8, 10, 11). Interestingly, most tumor-derived MDSCs are polymorphonuclear (12, 13). Although some important transcription factors and signaling pathways have been identified to regulate the differentiation of tumor-derived MDSCs (14–16), the concrete mechanisms remain to be fully elucidated.

IRF4, also known as LSIRF, ICSAT, Pip and Mum1, was first cloned independently as a member of the IRF gene family in 1996 (17). Under physiological conditions, IRF4 is a key regulator of the differentiation of lymphoid, myeloid and DC, including the differentiation of mature B cells into plasma cells (18). Recent studies have found that the abnormal expression of IRF4 is closely related to the occurrence of various malignant tumors (lymphoma, multiple myeloma, etc.) and autoimmune diseases (19, 20). Numerous studies suggest that IRF4 is an oncogene (21, 22), for instance, Weilemann et al. proposed that IRF4 is needed for the survival of anaplasia large cell lymphoma (21). Some studies also suggest that IRF4 is a tumor suppressor gene (23, 24). For example, Naresh et al. suggest that follicular lymphoma does not express or rarely expresses IRF4 (23). However, the function of IRF4 in tumor immunology is still poorly understood compared with the extensive studies on IRF4 in tumor biology (19). Recently, it has been reported that IRF4 can regulate differentiation in the myeloid system and DC cells (25, 26), the silencing of IRF4 could promote the development and function of MDSCs (27).

The c-Myc gene, a crucial member of the Myc gene family, is an adjustable gene, which could be regulated by a variety of substances. It regulates the transcription of thousands of genes required for a range of cellular processes, including proliferation, differentiation, and metabolism, which is closely related to the development of various tumors (28). In addition to the pivotal role in tumors, Myc is involved in physiological and pathological processes of many other immune diseases. Studies have confirmed that the expression of Myc family members in immune cells is strictly regulated during the development or activation of immune cells (29).

## Materials and Methods

### Ethics Statement

This research was approved by the Ethics Review Board of Guangzhou Medical University; written informed consent was provided by the study participants. All experimental protocols using animals were approved by the Animal Care and Use Committee of Guangzhou Medical University. Animal experiments were performed in strict accordance with the regulations of the Administration of Affairs Concerning Experimental Animals, and all efforts were made to minimize suffering.

### Mice and Cell Lines

IRF4 conditional (floxed) mutant mice *(IRF4^flox/flox^*; Stock No. 009380) and *LysM-Cre* mice (B6N.129P2 (B6) Lyz2tm1(cre)Ifo/J; Stock No: 018956) were originally were purchased from the Jackson Laboratory (Bar Harbor, ME, USA) and maintained with a C57B/L6 background. All mice were housed in a specific pathogen-free facility. All cell lines, including B16-F10 (B16), 3T3, 293T, and 32D were purchased from American type culture collection (ATCC). Female C57BL/6 mice were purchased from the Animal Experimental Center of Sun Yat-Sen University (Guangzhou, China).

### Generation of Interferon Regulatory Factor 4 KO Mice

IRF4 KO mice were generated as described previously (30). *LysM-Cre* mice were mated with *IRF4^flox/flox^* mice, and cohorts were established by mating F1 *IRF4^flox/+^*; *Cre^+^* mice to littermate *IRF4^flox/+^*; *Cre^-^* mice. The mice were maintained under a 14-h light/10-h dark cycle at a constant temperature (22°C) with free access to food and water.

### Reagents

The following reagents, including Concanavalin A (Con A), dimethyl sulfoxide and c-Myc inhibitor (10074-G5) were purchased from Sigma-Aldrich (St. Louis, MO). The recombinant mouse cytokines, including GM-CSF, IL-6, and IL-4 were obtained from Peprotech (Rocky Hill, NJ). The antibodies against IRF4, S100A9, c-Myc, and β-actin and HRP-conjugated secondary antibodies were purchased from Santa Cruz Biotechnology (Santa Cruz, CA). The following fluorescein-conjugated anti-mouse antibodies: Gr-1-PE-Cy7 (RB6-8C5), Gr-1-PE (RB6-8C5), Ly-6C-PerCP-Cyanine5.5 (HK1.4), CD11b-FITC (M1/70.15), CD11b-PE-Cy7 (M1/70.15), CD3e-FITC (145-2C11), CD4-PE (RM4-5), CD8a-PE-Cy5 (53-6.7), CD8a-PE-Cy7 (53-6.7), PD-L1–APC (MIH5), PD-L2–Brilliant Violet 421 (TY25), GM-CSF-PerCP-Cy5.5 (MP1-22E9), IL-1α–PE (ALF-161), IL-10–APC (JES5-16E3), and IL-6–APC (MP5-20F3) and the corresponding isotype antibodies as well as the anti-human antibodies CD33-PE (HIM3-4), CD11b-FITC (ICRF44), and HLA-DR-PE-Cy5 (L243) and their isotype control antibodies (QA16A12) were obtained from Biolegend (San Diego, CA). Fluorescein-conjugated anti-mouse antibody Ly-6G-PE (1A8) was purchased from BD Biosciences (San Jose, CA). Lipofectamine 2000, 5,6 carboxy fluorescein diacetate succinimidyl ester (CFSE) and the reagents for cell culture were purchased from Invitrogen (Carlsbad, CA). Mouse Ly6G microbeads were purchased from Miltenyi Biotec (Teterow, Germany).

### Microarray Analysis

An aliquot of 0.1 µg of total RNA was used to synthesize double-stranded cDNA, then produce biotin-tagged cRNA using the MessageAmp™ Premier RNA Amplification Kit. The resulting bio-tagged cRNA were fragmented to strands of 35–200 bases in length according to the protocols from Affymetrix. Hybridization was performed at 45°Cwith rotation for 16 h (Affymetrix GeneChip Hybridization Oven 640). The GeneChip arrays were washed and then stained (streptavidin-phycoerythrin) on an Affymetrix Fluidics Station 450 followed by scanning on a GeneChip Scanner 3000. The hybridization data were analyzed using GeneChip Operating software (GCOS 1.4). The scanned images were first assessed by visual inspection then analyzed to generate raw data files saved as CEL files using the default setting of GCOS 1.4. An invariant set normalization procedure was performed to normalize the different arrays using DNA-chip analyzer.

### Tumor Models and Analyses

To establish tumor growth models (31), B16-F10 tumor cells (1×10^5^) were injected subcutaneously (s.c.) into the flanks of mice. The tumors were measured every 2–3 days with calipers, and the volumes were calculated as V = ½ (length [mm] × [width {mm}]^2^). For tumor metastasis models (32), mice were injected intravenously with B16-F10 tumor cells (1×10^5^). At 3–4 weeks post tumor injection, the lungs were inflated with formalin followed by nodule counts and hematoxylin/eosin (H&E) staining.

### Myeloid-Derived Suppressor Cell Depletion

For PMN-MDSCs depletion (32, 33), anti-Ly6G antibodies (IA8; BD Biosciences) were injected (80 μg per injection) through the tail vein 3 days and 1 day before and 1 day after the injection of tumor cells. Depletion efficiency was evaluated by flow cytometry 3 weeks after the tumor injection. The anti-IgG antibody (BioLegend, San Diego, CA) was used as a control.

### *In Vitro* Generation of Myeloid-Derived Suppressor Cell

To generate MDSCs, we followed previously described procedures (34). Mouse Bone marrow (BM) cells were obtained from the femurs and tibias of mice and cultured in 24-well plates in RPMI 1640 medium containing 10% FBS, 50 mM 2-mercaptoethanol, 10 ng/ml IL-6, and 20 ng/ml GM-CSF. After 5 days of culture, the level of MDSCs was analyzed by flow cytometry. For MDSCs cultured with supernatant from tumor cells or 3T3 cells: BM cells from naive mice were cultured with GM-CSF and IL-6 in the presence of 30% (vol/vol) 3T3 or B16-F10 tumor supernatants (TS), After 2 days of culture, IRF4 expression was evaluated by qRT-PCR or by WB.

### Invasion Assay

Matrigel matrix solution (200 μg/ml, Matrigel™ Basement Membrane Matrix, BD Bioscience) was applied to each transwell (Falcon, Franklin Lakes, NJ, USA). B16 cells (5×10^4^) were seeded on the upper chamber of the transwell, and the lower chamber was then filled with collagen matrix (5 μg/ml). Noninvading cells on top of the matrix were removed after 18 h, and invading cells on the lower surface of the Matrigel matrix were fixed with 4% PFA and stained with 0.2% crystal violet. The cells were counted using ImageJ software (version 1.46).

### Cell Surface Staining

Cells were washed twice in sterile PBS (500 g, 8 min), and blocked in PBS containing 1% BSA for 30 min. Then, the cells were stained with conjugated antibodies that were specific for cell surface antigens for 30 min at 4°C in dark. These antigens included CD11b, Gr1, Ly6G, Ly6C, CD3e, CD4, CD8a, PD-L1, PD-L2, CD33, HLA-DR, CD14, and CD15. The stained cells were washed twice in in washing buffer (PBS containing 0.1% BSA), and re-suspended in 300ul washing buffer. Cells were analyzed by using flow cytometry (Beckman Coulter, Fullerton, CA), and the results were analyzed with use of the software CytoExpert 2.0 (Beckman Coulter). Isotype-matched cytokine controls were included in each staining protocol.

### Cell Sorting

For sorting of the mouse PMN-MDSC cells, mouse splenocytes were stained with CD11b-PE-Cy7, Ly-6G-PE, and Ly-6C-PerCP-Cyanine5.5 antibodies by cell surface staining as described before, and CD11b^+^Ly6G^+^Ly6C^−/low^ cells were isolated by cell sorting on a FACS Aria cell sorter (BD, Mountain View, CA). For sorting of the human PMN-MDSC cells, peripheral blood mononuclear cells were stained with CD33-PE, CD11b-FITC, and HLA-DR-PE-Cy5, and HLA-DR^-^CD11b^+^CD33^low^ cells were isolated by cell sorting on a FACS Aria cell sorter (BD, Mountain View, CA). The purified cells were identified by FACS, the purification of sorted cells was above 90%.

### Cell Intracellular Cytokine and Molecule Staining

Single-cell suspensions from the spleens of WT and IRF4 KO tumor bearing mice were stimulated with 20 ng/ml phorbol 12-myristate 13-acetate (PMA) plus 1 µg/ml ionomycin for 5 h at 37°C under a 5% CO2 atmosphere. Brefeldin A (10 g/ml, Sigma, Shanghai, China) was added during the last 4 h of incubation. Cells were washed twice in PBS, fixed with 4% paraformaldehyde, and permeabilized overnight at 4°C in PBS buffer containing 0.1% saponin (Sigma), 0.1% BSA, and 0.05% NaN3. Cells were then stained for 30 min at 4°C in the dark with conjugated antibodies specific for the cell surface antigens CD11b, and Gr1 as well as the intracellular cytokines or proteins GM-CSF, IL-10, IL-1α, and IL-6. The expression phenotypes of the antibody-labeled lymphocytes were analyzed by flow cytometry (Beckman Coulter, Fullerton, CA), and the results were analyzed with the software CytoExpert 2.0 (Beckman Coulter). Isotype-matched cytokine controls were included in each staining protocol.

### Lentivirus Transduction

The lentiviral stock preparation and viral transduction were performed as previously described (35). HEK 293T cells were transfected with lentiviral vectors and packaging plasmids (pCMV-ΔR8.2, pMD.G) using Lipofectamine 2000. The culture supernatants were collected, concentrated and stored at -80°C. BM cells were infected with a 30% volume of concentrated lentiviral stock solution (the virus titer was 2×10^8^ TU/ml) with 8 μg/ml polybrene. The medium was replaced with fresh medium at 3 h postinfection. The efficiency of infection was about 70%.

### Quantitative RT-PCR

The total RNA was extracted with an RNase Minikit, and cDNA was synthesized with SuperScript III reverse transcriptase (Qiagen, Valencia, CA). PCR was performed in triplicate using SYBR Green Mastermix (TaKaRa, Otsu, Japan) and was normalized to endogenous β-actin. The primer sequences used are listed in **Supplemental Table 1**.

### Western Blotting

Cultured or purified cells were collected and lysed. The protein concentration was measured with a bicinchoninic acid protein assay kit (Beyotime). The protein sample was separated in 10% SDS-denatured polyacrylamide gel and transferred to a polyvinylidene difluoride membrane. The polyvinylidene difluoride membranes were blocked with 5% skim milk in TBST at room temperature for 2 h. The targeted molecules were probed using specifc primary Abs and HRP-conjugated secondary Abs and were detected with an ECL HRP chemiluminescent substrate reagent kit (Invitrogen, Carlsbad, CA).

### Chromatin Immunoprecipitation Assay

The ChIP assay was performed following the instructions from Millipore (Billerica, MA, USA). In brief, cultured BM cells were fixed with a 1% formaldehyde solution, lysed and sheared by sonication. The cell lysates were precleared with protein-G-agarose and immunoprecipitated with specific antibodies or the anti-IgG control. The antibody-chromatin complexes were collected with protein-G-agarose. The DNA in the complex was recovered and quantitated with qPCR. As an input control, 10% of the lysate was used before immunoprecipitation. The amplification of cyclophilin from the input was used as a loading control.

### T-Cell Proliferation Assay

To quantify T-cell proliferation, we followed previously described procedures (35). Briefly, T-cell proliferation was determined by CFSE dilution. CD3^+^ T cells from BALB/c mice was Purified by flow cytometric sorting, and labeled with CFSE (1 μM) (Invitrogen), stimulated with concanavalin A (5 μg/ml) and cultured alone or co-cultured with allogeneic MDSCs (from WT or IRF4 KO mice) at different ratios for 3 days. The cells were then stained with CD4-PE or CD8-PE-Cy5 antibodies, and T-cell proliferation was analyzed by flow cytometry.

### Plasmid Constructs and Transfection Assays

The 5’-regulatory sequence of the mouse c-Myc gene was amplified by PCR using the primers listed in **Supplemental Table 1**. The wild type or mutated c-Myc promoter fragments were cloned into a pGL3-Basic vector (Promega), and the recombinations were confirmed by DNA sequencing. Transient transfections of the reporter plasmid were performed on 32D cells using Lipofectamine 2000 following the manufacturer’s instructions. The luciferase activity was measured at 48 h post transfection.

### Patients

Hepatocellular carcinoma (HCC) patients (n=20), individuals with hepatic fibrosis (n=20), were recruited at the Third Affiliated Hospital of Sun Yat-sen University (Guangzhou, China). Patients who had recently been pyrexial, had clinical evidence of an active infection, had previous or secondary cancers, or had received corticosteroids or nonsteroidal anti-inflammatory drugs were excluded from the study. The basic characteristics of patients are outlined in **Supplemental Table II**.

### Statistics

The data were analyzed using Mann-Whitney tests, χ^2^ tests, or Student’s t tests as appropriate. The correlations between different parameters were analyzed using a Spearman rank test. Statistical tests were performed using Graph Pad Prism version 5.0a and SPSS Statistics 17.0. P-values of less than 0.05 were considered significant.

## Results

### Decreased Interferon Regulatory Factor 4 Expression in Tumor-Deriving Myeloid-Derived Suppressor Cells

**Figure 1:**
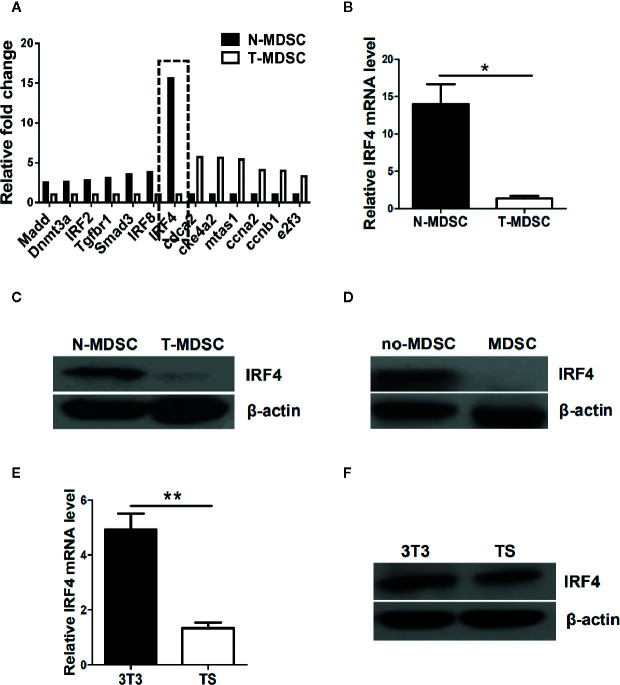
Interferon regulatory factor 4 (IRF4) expression decreases in tumor-derived MDSCs. **(A)** Microarray analysis showing differentially expressed genes in splenic myeloid-derived suppressor cells (MDSCs) from tumor-bearing mice was injected with B16-F10 cells *via* the subcutaneously (T-MDSC) and the corresponding control cells from naive mice (N-MDSC). **(B)** Interferon regulatory factor 4 (IRF4) was evaluated by qRT-PCR with additional samples. **(C)** IRF4 expression in splenic MDSCs from tumor-bearing mice and control cells from naive mice was determined by a western blot (WB). **(D)** IRF4 expression in splenic CD11b^+^Gr1^+^cells and CD11b^+^Gr1^-^cells from tumor-bearing mice was determined by WB. **(E, F)** Bone marrow (BM) cells from naive mice were cultured with GM-CSF and IL-6 in the presence of 30% (vol/vol) 3T3 or B16-F10 tumor supernatants (TS); IRF4 expression was evaluated by qRT-PCR (e) and WB (f). **(B, E)** Data are shown as the mean ± SEM of six samples from three independent experiments. *P < 0.05, ** *P* < 0.01 compared with the corresponding controls in unpaired t tests.

To determine the potential regulatory mechanism of MDSCs in tumor, a melanoma B16-F10 (B16) was used to establish a tumor mouse model. Gene chips were analyzed and screened by using MDSCs (T-MDSCs) sorted from tumor-bearing mouse spleens with immature myeloid cells from normal mouse spleens (N-MDSCs) as a control. We found that the expression of interferon regulatory factor 4 (IRF4) in the MDSCs of the tumor group was significantly down-regulated (**Figure 1A**). This result was validated by qRT-PCR (*P*<0.05, **Figure 1B**). The western blot (WB) further confirmed that expression of IRF4 in T-MDSCs was clearly down-regulated compared with N-MDSCs (**Figure 1C**). A lower expression of IRF4 was found in CD11b^+^Gr1^+^cells (MDSC) compared with CD11b^+^Gr1^-^ cells (no-MDSC) in the spleen of tumor-bearing mice (**Figure 1D**). *In vitro* cell culture showed that the expression of IRF4 in MDSCs induced by the supernatant of cultured tumor cells was significantly decreased compared with the MDSCs induced by the supernatant of cultured 3T3 cells (*P*<0.05, **Figures 1E, F**). These data demonstrated that lower level of IRF4 was expressed in the tumor-induced MDSCs. This finding suggested that IRF4 may be a key transcription factor regulating MDSCs differentiation and accumulation in tumor development.

### Interferon Regulatory Factor 4 Deficiency Could Facilitate Tumor Growth and Metastasis

**Figure 2:**
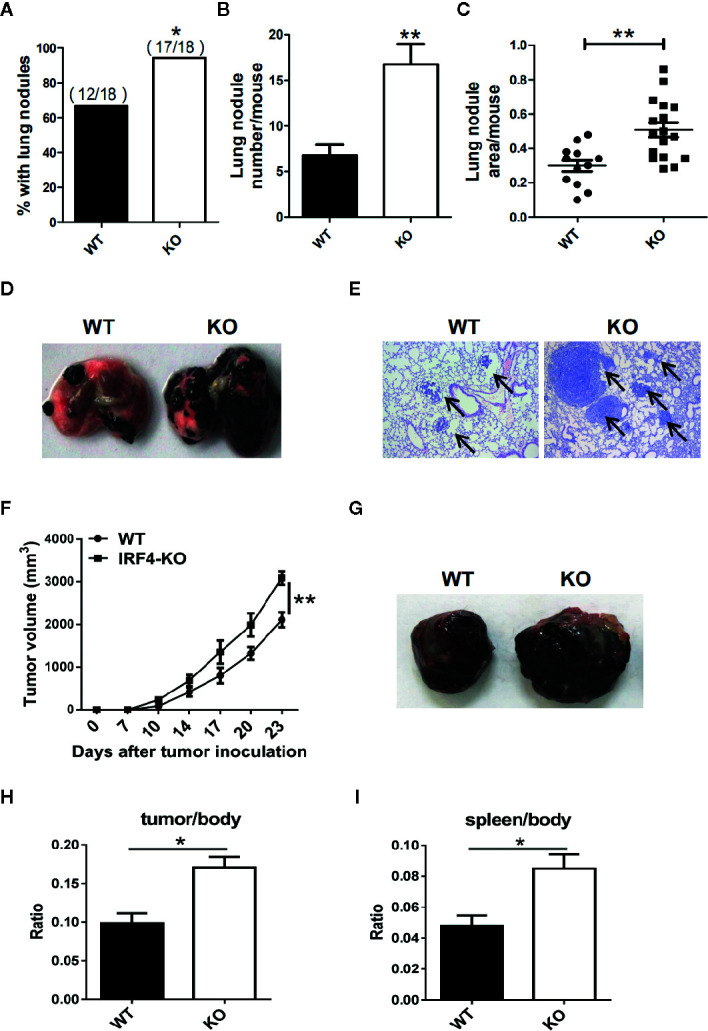
Interferon regulatory factor 4 (IRF4) deficiency in the host facilitates tumor development. **(A–E)** WT (n=18) or IRF4 KO (n=18) mice were injected with B16-F10 cells *via* the tail vein; mice were sacrificed after 3 weeks. **(A)** Percentage of mice with lung nodules; *P < 0.05, *χ^2^* test. **(B)** The number of lung nodules per mouse; *P < 0.05, Student’s *t* test. **(C)** Lung nodule area per mouse using NIH ImageJ; ***p < 0.001, Mann-Whitney test. **(D)** Representative images of lungs. **(E)** Representative images of lung H&E staining; arrows indicate metastases. **(F–I)** Tumor growth model; mice were subcutaneously injected with 1×10^5^ B16-F10 tumor cells (n=6). Primary tumor growth was monitored **(F)**; *P < 0.05, Mann-Whitney test. Representative images of tumor **(G)**. The ratio of tumor **(H)** or spleen **(I)** weight to mouse body weight; *P < 0.05, Student’s *t* test.

To investigate whether the IRF4 gene can affect tumor progression in mice, a tumor growth models and tumor metastasis models were established in mouse. 6–8 weeks old *IRF4^flox/flox^/LysM-Cre^+^* (IRF4 KO) female mice were selected for experiments, *IRF4^flox/flox^/LysM-Cre^-^* female mice (WT) of the same age as a control. To detect tumor metastasis, B16 cells were injected into the WT and IRF4 KO mice *via* the tail vein, and the status of the tumor metastasis was determined 3 weeks later. The number of lung tumor metastasized mice increased significantly compared with WT mice (*P*<0.05, **Figure 2A**). Moreover, the appearance of lung was imaged (**Figure 2D**), and the slice of lung tissues was stained by H&E staining and observed under microscope (**Figure 2E**). Result showed that the number of lung metastasis nodules in the mouse and the area of lung metastasis nodules in IRF4-deficient mice were significantly increased relative to the control (*P*<0.05, **Figures 2B, C**). These results indicated that absence of IRF4 could significantly promote lung tumor metastasis.

In addition, to detect the role of IRF4 in tumor growth, B16 tumor cells were injected into the WT and IRF4 KO mice subcutaneously. The diameter of tumor was recorded, and the mean volume of tumor was calculated from day 7 to day 23, with 3–4 days interval. The results showed that the volume of tumor in the skin of IRF4 KO was bigger than that in the WT mice on day 17, day 20, and day 23 (*P*<0.05, **Figure 2F**). Furthermore, 3 weeks after B16 injection, the spleens and tumors tissue (**Figure 2G**) were picked out from mice, and weighed. The body weight of WT and IRF4 KO mice were also detected. The ratio of tumor-to-body weight and spleen-to-body weight were calculated, respectively. The results showed that the weight ratios of tumor/body and spleen/body were significantly increased in the IRF4 KO mice (*P*<0.05, **Figures 2H, I**). These results suggested that a deficiency of IRF4 in tumor-bearing mice not only promote lung tumor metastasis, but also promote tumor growth significantly.

### Interferon Regulatory Factor 4 Inhibits the Effect of Primarily Polymorphonuclear-Myeloid-Derived Suppressor Cells on Tumor Growth and Metastasis

**Figure 3:**
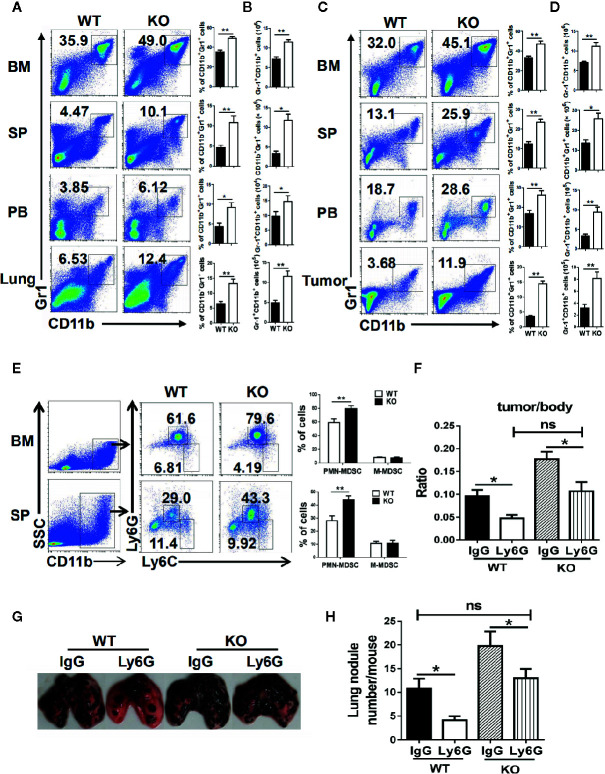
Interferon regulatory factor 4 (IRF4) deficiency causes polymorphonuclear myeloid-derived suppressor cells (PMN-MDSCs) elevation in tumor-bearing mice. **(A–D)** B16-F10 tumor cells were injected into WT or KO mice (n=6) *via* the tail veil to establish tumor metastasis **(A, B)** or tumor-growth **(C, D)** models. Mice were sacrificed after 3 weeks. The percentages **(A, C)** and absolute numbers **(B, D)** of MDSCs were analyzed by flow cytometry; *P < 0.05, **P < 0.01, Student’s t test. **(E)** The proportions of the MDSCs subtypes in the bone marrow (BM) and spleens from tumor-growth models were evaluated by flow cytometry. Each group included six mice; representative results (left) and the graphical representation (right) are shown; **P < 0.01, Student’s *t* test. **(F–H)** Mice (n=5) were injected intravenously with anti-Ly6G antibodies or an anti-IgG control before and after B16 tumor cell injection. **(F)** The ratio of tumor weight to mouse body weight; *P < 0.05, Student’s *t* test. **(G, H)** Lung transfer was evaluated 3 weeks after tumor injection. **(G)** Representative images of lung tissue. **(H)** The number of lung nodules per mouse; **P < 0.01, Student’s *t* test.

To detect the effect of PMN-MDSC on tumor growth and metastasis, B16 cells were injected to both WT and IRF4 KO mice from the vain of tail (tumor metastasis model) or subcutaneously (tumor growth models), respectively. Three weeks later, bone marrow (BM), spleen (SP), lung, peripheral blood (PB), and tumor tissue were picked out from B16-bearing WT and IRF4 KO mice. Mononuclear cells were isolated, respectively. The percentage of MDSCs (CD11b^+^ Gr1^+^) was analyzed by FACS. Results showed that the proportion and absolute number of MDSCs in samples from the IRF4 KO mice were significantly increased in both the tumor metastasis models (*P*<0.05, **Figures 3A, B**) and tumor growth models (*P*<0.05, **Figures 3C, D**).

Moreover, the subsets of MDSCs in KO tumor-bearing WT and IRF4 mice were also explored by FACS. As showed in **Figure 3E**, the percentage of CD11b^+^Ly6G^+^Ly6C^−/low^ PMN-MDSCs in the bone marrow and spleen of IRF4 KO tumor-bearing mice were increased significantly *(P*<0.01), whereas there was no significant change in the percentage of CD11b^+^Ly6G^−^Ly6C^high^ M-MDSCs (*P>*0.05). These findings suggested that IRF4 deletion can specifically result in the accumulation of PMN-MDSCs in the bone marrow and spleen of tumor mice.

To determine whether tumor progression was mediated by PMN-MDSCs mice, B16 cells were injected to both WT and IRF4 KO mice through the vain of tail or subcutaneously. Anti-Ly6G antibodies were injected into mice through the tail vein 3 days and 1 day before and 1 day after the injection of B16 cells as described in materials and methods. Three weeks later, the radio of the tumor weight to the body weight was calculated, and the number of lung nodule was counted. The results showed that anti-Ly6G antibodies could decrease the value of these two detections in both WT and IRF4 KO mice (*P*<0.05, **Figures 3F–H**). More interesting is that the elimination of PMN-MDSCs can clearly reverse tumor growth (**Figure 3F**) and lung tumor metastasis (**Figures 3G, H**) in IRF4 KO mice. These data indicated that IRF4 mediates the effect of PMN-MDSCs on tumor growth and metastasis.

### Interferon Regulatory Factor 4 Deficiency Enhance the Immunosuppressive Function of Primarily Polymorphonuclear-Myeloid-Derived Suppressor Cells

**Figure 4:**
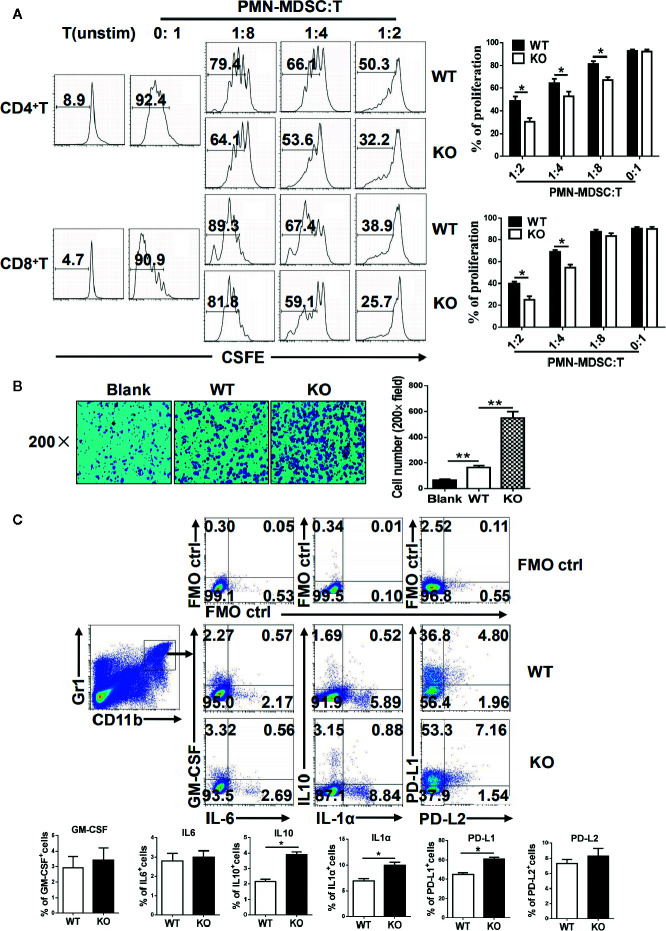
Functional analysis of myeloid-derived suppressor cells (MDSCs). **(A)** Allogeneic mixed lymphocytes reaction. Allogeneic CD3^+^ T cells were stimulated with concanavalin A (ConA) and then cocultured with splenic G-MDSCs that were purified with Ly6G beads from the spleen of tumor-bearing mice at different ratios for 3 days. T-cell proliferation was evaluated by CFSE dilution; unstimulated T cells were used as a negative control. Representative data from single experiment (left) and mean ± SEM from three independent experiments (right) are shown. **(B)** B16 cells were cocultured with polymorphonuclear (PMN)-MDSCs, and a cell invasion assay was performed with Matrigel (crystal violet). Left, representative from a single experiment; right, mean ± SEMs from three independent experiments, *P < 0.05, **P < 0.01, unpaired *t* test. **(C)** Single-cell suspensions of spleen cells from WT and IRF4 KO tumor-bearing mice were stimulated with PMA and ionomycin. The expression of PD-L1, PD-L2, GM-CSF, IL-1a, IL-6, and IL-10 were detected in MDSCs by FACS. Numbers in the quadrants are the percentages of cells in each expression phenotype (n = 5 mice per group). A representative of two independent experiments is shown.

MDSCs are characterized by their immunosuppressive function, and we next investigated whether an IRF4 deficiency could influence the function of PMN-MDSCs. The splenic PMN-MDSCs from tumor-bearing WT and IRF4 KO mice were sorted by flow cytometry and mixed with T lymphocytes derived from allogeneic mice in different ratios (stimulated by ConA and labeled with CFSE). Three days later, the proliferation of T cells was detected by FACs. The results showed that PMN-MDSCs from the IRF4 KO group had a stronger ability to inhibit T cell proliferation than WT-derived PMN-MDSCs *(P*<0.05, **Figure 4A**). Simultaneously, a difference in tumor metastasis was detected in WT and IRF4 KO tumor-bearing mice. In the tumor invasion experiment, B16 tumor cells were co-cultured with PMN-MDSCs derived from the spleens of both WT and IRF4 KO mice for 18 h. The results demonstrated that the PMN-MDSCs derived from IRF4 KO mice possessed a greater ability to promote tumor invasion compared with those from the WT group (*P* < 0.01, **Figure 4B**). The ability of MDSCs in producing inflammatory factors, including IL-1a, IL-6, IL-10, and GM-CSF, and the expression of PD-L1 and PD-L2 (programmed cell death 1 ligand 1/2) were detected by flow cytometry. As showed in **Figure 4C**, the significantly higher levels of IL-1a and IL-10 producing MDSCs were found in IRF4 KO mice (*P*<0.05) compared with the WT mice, whereas there was no clear difference in GM-CSF and IL-6 production (*P>*0.05). Additionally, the expression of PD-L1 on MDSCs derived from IRF4 KO mice was significantly higher than that from WT mice. There was also no difference between the groups in the expression of PD-L2 on MDSCs, (P>0.05, **Figure 4C**). These results revealed that an IRF4 deficiency could enhance the immunosuppressive function of PMN-MDSCs in both tumor growth and tumor invasion, and enhance the ability of MDSCs to produce inflammatory factors.

### c-Myc Mediate the Effects of Interferon Regulatory Factor 4 on Myeloid-Derived Suppressor Cell Development

**Figure 5:**
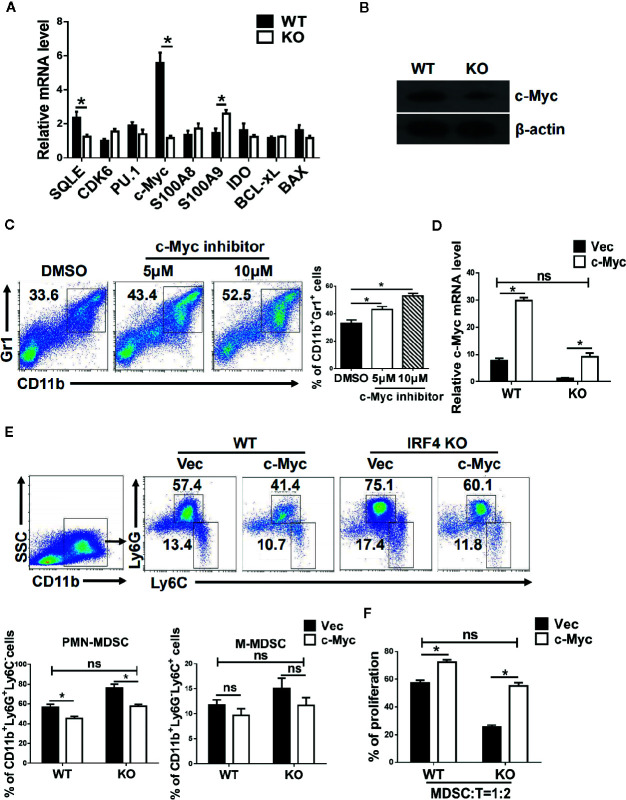
c-Myc mediates the effects of interferon regulatory factor 4 (IRF4) on myeloid-derived suppressor cells (MDSCs) development. **(A, B)** Gene expression in sorted MDSCs was determined by quantitative RT-PCR (qRT-PCR) **(A)** and western blot **(B)**. **(C)** Mouse bone marrow (BM) cells from normal mice were cultured in medium containing GM-CSF and IL-6 with the different concentrations of c-Myc inhibitor (10074-G5). The proportions of the indicated populations were determined by flow cytometry after 5 days of culture. **(D–F)** BM cells from WT or KO mice were infected with lentivirus expressing c-Myc or an empty vector. **(D)** The c-Myc gene expression was determined by qRT-PCR after 48 h of culture. **(E)** The proportions of indicated populations were determined by flow cytometry after 5 days of culture. **(F)** MDSCs were purified by flow cytometric sorting. Allogeneic CD3^+^ T cells (from BALB/c mice) were stimulated with Con A and then co-cultured with isolated PMN-MDSCs at 2:1 ratios for 3 days. T cell proliferation was evaluated by 5,6 carboxy fluorescein diacetate succinimidyl ester (CFSE) dilution. A comparison of the suppressive activity on CD3^+^ T cells between MDSCs from WT or KO mice were infected with lentivirus expressing c-Myc or an empty vector. **(A, D, F)** Data are shown as the mean ± SEMs from three independent experiments. *P < 0.05, compared with the corresponding controls; unpaired t tests were used. **(C, E)** Representative results (left) and mean ± SEMs from 3 independent experiments; *P < 0.05, unpaired *t* tests.

To explore the mechanism by which IRF4 regulates PMN-MDSCs differentiation and tumor metastasis, the potential target genes of IRF4 in MDSCs and the genes related to the differentiation and survival of MDSCs were detected by gene expression. As showed in **Figure 5A**, the gene expression and protein expression levels of c-Myc in MDSCs derived from IRF4 KO mice were significantly down-regulated compared with the MDSCs derived from WT mice (*P*<0.05). Next, the expression of c-Myc protein in MDSCs was detected by the method of western blotting. Results showed that the expression of c-Myc protein in MDSCs derived from IRF4 KO mice was decreased significantly (*P*<0.05, **Figure 5B**). Moreover, different concentrations of c-Myc inhibitors were added to the cultured MDSCs *in vitro* to confirm the effects of c-Myc on the differentiation of MDSCs. The results indicated that c-Myc inhibitors increased the proportion of MDSCs in a concentration-dependent manner (**Figure 5C**). Additionally, a lentivirus containing a c-Myc over-expression plasmid was added to cultured bone marrow cells from both WT and IRF4 KO mice to induce MDSCs *in vitro* for 5 days. As showed in **Figures 5D–F**, results indicated that c-Myc over-expression in bone marrow cells (**Figure 5D**) and decreased percentage of MDSCs induced by IRF4 deletion could be produced by over-expression of c-Myc (**Figure 5D**). Furthermore, c-Myc over-expression significantly increased the suppressive activity of MDSCs derived from IRF4-deficient cells (**Figure 5F**). These results suggested that c-Myc may mediate the effects of IRF4 on MDSCs development and function.

### c-Myc is a Transcriptional Target of Interferon Regulatory Factor 4 in Myeloid-Derived Suppressor Cells

**Figure 6:**
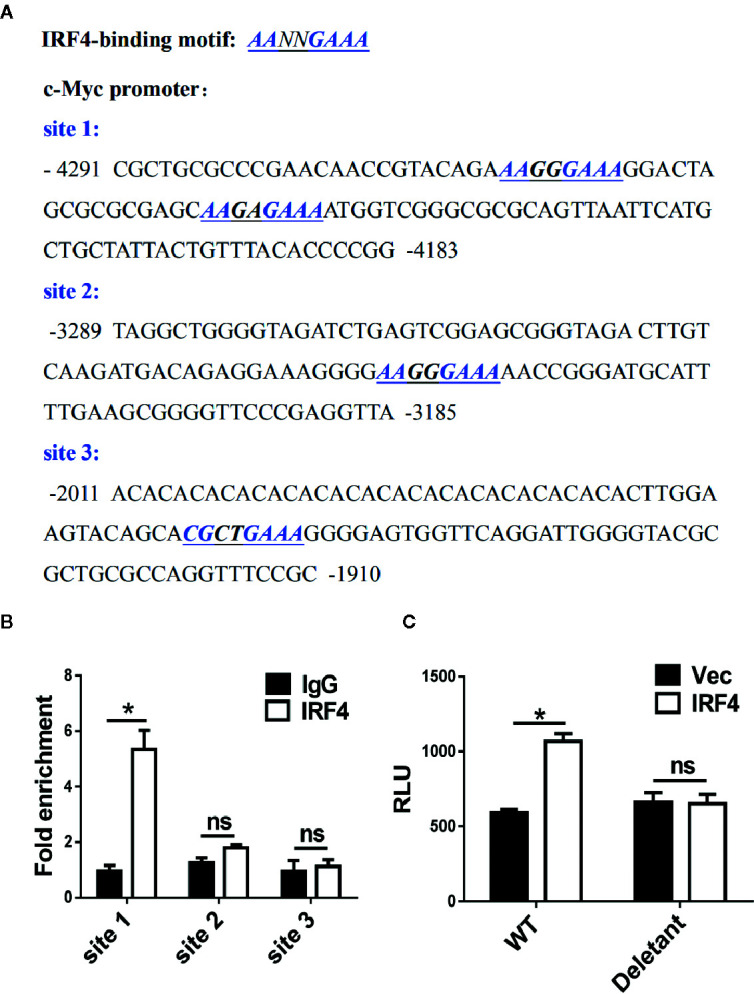
c-Myc is a transcriptional target of interferon regulatory factor 4 (IRF4) in myeloid-derived suppressor cells (MDSCs). **(A)** Sequence analysis of c-Myc promoter; the potential IRF4-binding sites are underlined. **(B)** A chromatin immunoprecipitation (ChIP) assay was performed on a 3-day culture of bone marrow (BM) cells using anti-IRF4 or anti-IgG antibodies; the presence of the c-Myc promoter harboring the potential IRF4 binding sites (site 1: −4,291~−4,183) was measured by qPCR. Site 2 (−3,289~−3,185) and site 3 (−2,011~−1,910) were detected in parallel as controls. The data were normalized against input and presented as the fold increase over the IgG control. **(C)** 32D cells were co-transfected with the c-Myc reporter (WT, +157 ~−4,480) or deletant (+157 ~−3,573) and the plasmid expressing IRF4 or vector; luciferase activity was measured 48 h posttransfection. **(B, C)** Mean ± SEMs from three independent experiments; *P < 0.05, ns *P* > 0.05, unpaired t tests.

The mechanism of c-Myc regulation by IRF4 in MDSCs was further investigated in the tumor microenvironment. First, a potential IRF4 binding site was identified in the regulatory region of c-Myc (near the region from −4,183 to −4,291 bp upstream of the transcription start site) after screening (**Figure 6A**). The chromatin immunoprecipitation experiments confirmed that the IRF4 protein can bind to these two sites (**Figure 6B**). Further experiments demonstrated that the over expression of IRF4 in the 32D myeloid cell line promoted the activity of c-Myc (*P*<0.05), but this effect disappeared when the potential binding site of IRF4 was deleted (**Figure 6C**). These results demonstrated that IRF4 regulates the expression of c-Myc at the level of transcription.

### Clinical Significance of Interferon Regulatory Factor 4 Regulated Primarily Polymorphonuclear-Myeloid-Derived Suppressor Cells Development

**Figure 7:**
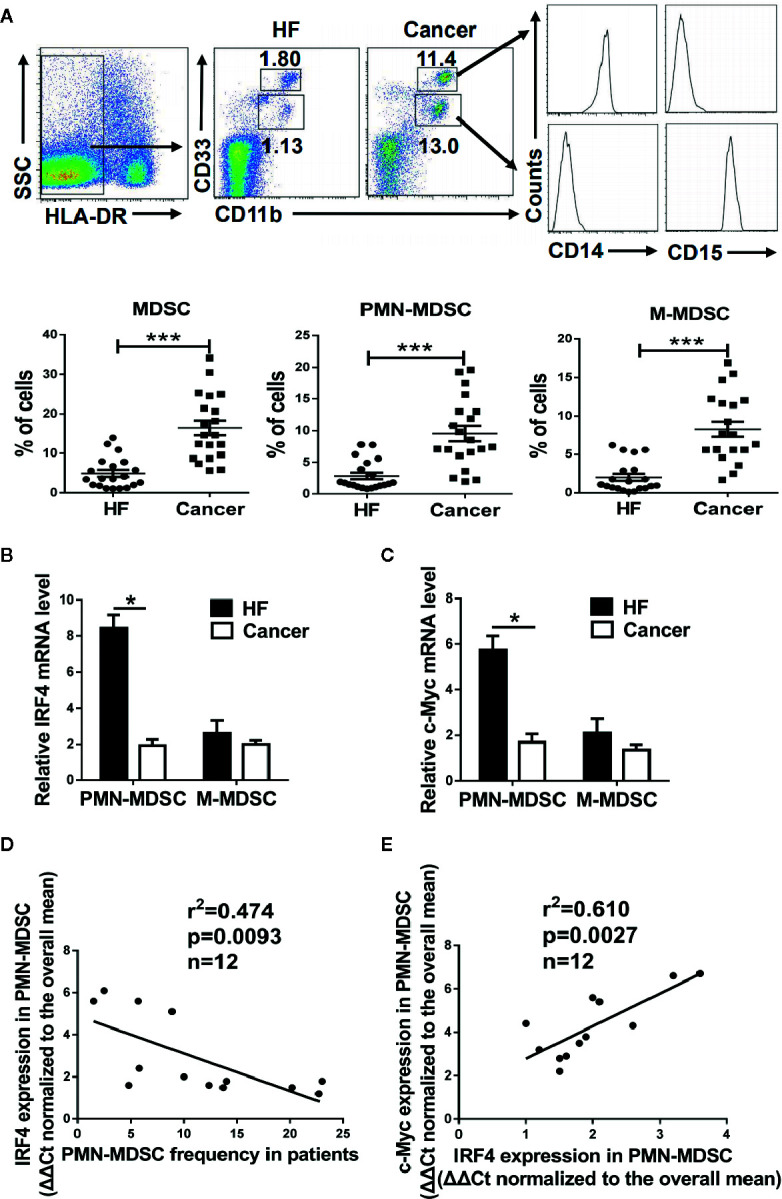
Clinical significance of interferon regulatory factor 4 (IRF4)-mediated polymorphonuclear myeloid-derived suppressor cells (PMN-MDSCs) development. Peripheral blood samples were collected from hepatocellular carcinoma (HCC) patients (n=20); individuals with hepatic fibrosis (HF) (n=20) were used as a control. The levels of MDSCs and their subsets were determined by flow cytometry. **(A)** Representative results (upper) and mean ± SEMs (lower) are shown. **(B, C)** The expression of IRF4 **(B)** and c-Myc **(C)** in PMN-MDSCs and M-MDSCs were determined by qRT-PCR. Mean ± SEMs from 4 individuals are shown. **(D, E)** Correlations between IRF4 expression and PMN-MDSCs frequency (n=12) **(D)** and c-Myc expression in PMN-MDSCs (n=12) **(E)** are shown; Spearman rank test.

To explore the clinical significance of IRF4-mediated differentiation of PMN-MDSCs, peripheral blood samples from patients with liver cancer (HCC) were collected, and peripheral blood samples from nontumor patients with liver fibrosis served as controls. The proportion of the M-MDSCs (HLA-DR^-^CD11b^+^CD33^hi^CD14^+^) and PMN-MDSCs (HLA-DR^-^CD11b^+^CD33^low^ CD15^+^) in the peripheral blood of liver cancer patients was significantly increased (*P*<0.01, **Figure 7A**). Moreover, the expressions of IRF4 and c-Myc in PMN-MDSCs and M-MDSCs from tumor patients was explored. Results showed that the expressions of IRF4 and c-Myc were down-regulated in PMN-MDSCs from tumor patients compared with those in the controls (*P*<0.05), but no significant change was detected in expression of IRF4 in M-MDSCs (**Figures 7B, C**). Furthermore, the expression of IRF4 in PMN-MDSCs was inversely correlated with the proportion of PMN-MDSCs in liver cancer patients (**Figure 7D**). Consistent with the experimental results in mice, the expression of IRF4 was also positively correlated with the gene expression of c-Myc in the PMN-MDSCs from tumor patients (**Figure 7E**). These results indicated that IRF4 mediated PMN-MDSCs differentiation has very important clinical significance during tumor progression.

## Discussion

Myeloid-derived suppressor cells (MDSCs) has well known roles in the suppression of anti-tumor immunity in tumor-bearing hosts (2, 3). However, few reports have focused on the mechanisms controlling the development and differentiation of MDSCs (33, 36, 37). Therefore, elucidation of the signaling events controlling MDSCs subsets will facilitate the development of an efficient MDSC-based clinical therapy.

It has been reported that IRF4 can regulate differentiation in the myeloid system and DC cells (25, 26), the silencing of IRF4 could promote the development and function of MDSCs (27). However, the role in the lineage determination of immune cells remains unknown. Despite the extensive studies on the roles of IRF4 in tumor biology, the function in tumor immunology remains poorly understood. Under physiological conditions, the regulatory role of IRF4 in myeloid cell differentiation deserves further investigation. Here, we demonstrate that IRF4 represents a novel regulator of PMN-MDSCs, but not of M-MDSCs and IRF4 expression is also negatively correlated with PMN-MDSCs levels in clinical HCC patients. Thus, our results indicate that IRF4 may play an important role in MDSCs subset determination.

IRF4 has been shown to be important for efficient antigen cross-presentation of moDC (38), and IRF4 expression cloud induce macrophage by cytokines activation and polarization (39). Given the substantial reduction of moDC cells and induction of M2 cells, it seems more likely that this is due to an impaired sustained activation of anti-tumoral T cells than to the amplification and action of MDSC in IRF4-KO mice. We demonstrated that the PMN-MDSC frequency was correlated with tumor weight and metastasis in the B16 model. It suggested that the elevated levels of PMN-MDSC in the IRF4 KO mice could be a secondary effect of the increased tumor progress.

Valdez et al. reported that Prostaglandin E2 can suppress IRF4 expression in T cells (40). Meanwhile, Prostaglandin E2 promotes tumor progression by inducing myeloid-derived suppressor cells (41). These studies suggest a possibility that a high level of prostaglandin E2 in the tumor microenvironment induces MDSCs development by suppressing IRF4 expression. Here, we found that the expression of IRF4 was decreased in the MDSCs treated with supernatant from tumor cells compared with the supernatant from 3T3 cell. It implied that there might be some Prostaglandin E2 in the supernatant of cultured tumor cells which decreased the expression of IRF4 in MDSCs. Further experiment was needed to elucidate it.

Although the existing evidence suggests that Myc family members play a crucial role in regulating the development, differentiation and activation of immune cells (macrophages, dendritic cells, B cells and T cells, etc.) (42, 43), no studies have focused on the regulation of MDSCs differentiation and function by the c-Myc gene. In this study, the important role of the c-Myc gene in regulating the differentiation and function of MDSCs is elucidated and can be targeted for MDSCs treatment. These findings provide a new and important theoretical and experimental basis for improving the efficacy of current tumor immunotherapy.

MDSCs expansion in human tumors has also been extensively studied, revealing that MDSCs derived from distinct types of tumors vary with respect to both their phenotype and immune properties (8). Regardless, the significance of MDSCs subsets in clinical cancer patients is not well defined. In this study, we found that PMN-MDSCs, but not M-MDSCs, are associated with tumor metastasis in HCC patients. The negative correlation between IRF4 expression and PMN-MDSCs levels further supports the pathological significance of IRF4 in PMN-MDSCs development. However, we would like to note that the relationship between IRF4 and PMN-MDSCs in human tumors requires further detailed investigation in distinct tumor types before firm conclusions can be drawn.

In conclusion, our study demonstrates that IRF4 is a novel regulator of PMN-MDSCs in cancer and that c-Myc is the transcriptional target of IRF4 in MDSCs. IRF4 may have predictive value for determining the PMN-MDSCs level and tumor progression in cancer patients.

## Data Availability Statement

The raw data supporting the conclusions of this article will be made available by the authors, without undue reservation.

## Ethics Statement

The studies involving human participants were reviewed and approved by the Ethics Review Board of Guangzhou Medical University. The patients/participants provided their written informed consent to participate in this study. The animal study was reviewed and approved by the Animal Care and Use Committee of Guangzhou Medical University. Written informed consent was obtained from the owners for the participation of their animals in this study. Written informed consent was obtained from the individual(s) for the publication of any potentially identifiable images or data included in this article.

## Author Contributions

QY and HX performed most of the experiments and analyzed the data with assistance from JH. XL collected the clinical samples, and YF, SX, JQ, AX, YZ, and LZ performed the animal experiment. JY, XH, and HW performed the selected immunoblots. HQ and WQ contributed to the scientific planning. QY and JH oversaw and designed the study. WQ, QY, and JH authored the manuscript. All authors contributed to the article and approved the submitted version.

## Funding

This work was supported by a grant from the Natural Science Foundation of China (31800739, 81970771, 81771696), the Natural Science Foundation of Guangdong Province (2018A0303130317), the Guangdong Provincial Education Department (2017KTSCX157), the Medical Research Fund of Guangdong Province (A2019471), and the Youth Project Fund of the State Key Laboratory of Respiratory Diseases (SKLRDQN-201921).

## Conflict of Interest

The authors declare that the research was conducted in the absence of any commercial or financial relationships that could be construed as a potential conflict of interest.

## Abbreviations

BM, bone marrow; CFSE, 5,6 carboxy fluorescein diacetate succinimidyl ester; ChIP, Chromatin immunoprecipitation assay; Con A, Concanavalin A; c-Myc, Cellular myelocytomatosis oncogene; HCC, Hepatocellular carcinoma; IRF, interferon regulatory factor; KO, knockout mice; LLC, Lewis lung carcinoma; M, monocytic; MDSCs, myeloid-derived suppressor cells; PMN, polymorphonuclear; PB, peripheral blood; SP, spleen; WT, wild-type C57BL/6 mice.
