# Supplementary material for: Interferon Regulatory Factor 4 Regulates the Development of Polymorphonuclear Myeloid-Derived Suppressor Cells Through the Transcription of c-Myc in Cancer
Source: Front Immunol. 2021 Feb 23;12:627072. doi: 10.3389/fimmu.2021.627072 (PMC7940347; doi:10.3389/fimmu.2021.627072)
Supplement: Supplementary file 1 [file DataSheet_1.pdf]

Supplementary Materials

Table I: Sequences of primers used in this study.

Primer	Sequence
IRF4-for	5′-AAACCATAGAGGCACCCAAG-3′
IRF4-rev	5′-CCCAATAGCCAGTCTCCAAA-3′
S100A8-for	5′-GGAAATCACCATGCCCTCT-3′
S100A8-rev	5′-TTTATCACCATCGCAAGGAAC-3′
S100A9-for	5′-AATGGTGGAAGCACAGTTGG-3′
S100A9-rev	5′-GCTGATTGTCCTGGTTTGTG-3′
CDK6-for	5′-GTTTTCAGATGGCCCTTACC-3′
CDK6-rev	5′-AAAGATGCAACCGACACTCC-3′
SQLE-for	5′-AGAGCCCGACAGGATAGTTG-3′
SQLE-rev	5′-TTCCACTCTGCACTTGGTTG-3′
PU.1-for	5′-ATGCACGTCCTCGATACTCC-3′
PU.1-rev	5′-CTCCAAGCCATCAGCTTCTC-3′
Arg1-for	5′-ATTATCGGAGCGCCTTTCTC-3′
Arg1-rev	5′-ACAGACCGTGGGTTCTTCAC-3′
IDO-for	5′-TCTGCTGTATGAGGGGGTCT-3′
IDO-rev	5′-GCCAGCCTCGTGTTTTATTC-3′
c-Myc-for	5′-CTGTACCTCGTCCGATTCCA-3′
c-Myc-rev	5′-TCTCCTCATGCAGCACTAGG-3′
Bcl-xL-for	5′-CGTGGAAAGCGTAGACAAGG-3′
Bcl-xL-rev	5′-GCTGCATTGTTCCCGTAGAG-3′
BAX-for	5′-GAGACACCTGAGCTGACCTT-3′
BAX-rev	5′-GTCCACGTCAGCAATCATCC-3′
β-actin-for	5′-TACCACAGGCATTGTGATGG-3′

β-actin-rev	5′-TTTGATGTCACGCACGATTT-3′
c-Myc-reporter-WT-for	5′-GGGGTACCCTCCTAGATAACTCATTCGTTCGTC-3′
c-Myc-reporter-Del-for	5′-GGGGTACCAACTTACAATCTGCGAGCCAGGACA-3′
c-Myc-reporter-rev	5′-CCCCCGGGGCGTAGTTGTTCTGGTGAGTGGAGA-3′
ChIP-site-1-for	5′-CATGGCGTATTGTGTGGAGC -3′
ChIP-site-1-rev	5′-GCTCCGGGGTGTAAACAGTA -3′
ChIP-site-2-for	5′-GAGTAGTTGGGGTAGGCTGG-3′
ChIP-site-2-rev	5′-TCAGCCCATAGTAACCTCGG-3′
ChIP-site-3-for	5′-ACACACACACACACACTTGG-3′
ChIP-site-3-rev	5′-CACAGCAAGGGAAGCAAGTC-3′
c-Myc-cDNA-for	5′- ATCGACCGGTCAAGAAGATGAGGAAGAAATTGAT-3′
c-Myc-cDNA-rev	5′-ATCGGCTAGCGAGAGATTCCAGCTCCTCCTCGAGT-3′
Hu-IRF4-for	5′-ATGACAACGCCTTACCCTTC-3′
Hu-IRF4-rev	5′-GTCACCTGGCAACCATTTTC-3′
Hu-c-Myc-for	5′-AAAAGGCCCCCAAGGTAGTT-3′
Hu-c-Myc-rev	5′-TCCGTAGCTGTTCAAGTTTGTG-3′
Hu-β-actin-for	5′-GCTCTTTTCCAGCCTTCCTT-3′
Hu-β-actin-rev	5′-CATACAGGTCTTTGCGGATGT-3′

Note: All the primers are designed for mouse genes, except indicated as Hu (human).

Table II: Basic characteristics of 20 HCC patients and 20 patients with hepatic fibrosis

Characteristics	HCC Patients (n=20)	hepatic fibrosis (n=20)	*P*
Gender			0.946
Male	17 (85.0%)	18 (90.0%)	
Female	3 (15.0%)	2 (10%)	

Age (years)	54.8 (32.4-76.9)	48.3 (18.9-72.3)	0.96
Etiology			0.59
Hepatitis B	15 (75%)	12 (60%)	
Other	5 (25%)	8 (40%)	
Portal vein thrombosis	12 (60%)	NA	NA
Lymph node metastases	7 (35%)	NA	NA
Distant Metastasis	1 (5%)	NA	NA
TNM 7th Edition			NA
I	6 (30%)	NA	
II	4 (20%)	NA	
III	9 (45%)	NA	
IV	1 (5%)	NA	
Child-Pugh Grade			0.478
A	10 (50%)	11 (55%)	
B	7 (35%)	5 (25%)	
C	3 (15%)	4 (20%)	

Note: HCC, hepatic carcinoma. *P*： *P* value, statistical tests were performed using SPSS Statistics 17.0.

a
